# The Role of the Insulin-Like Growth Factor 1 Pathway in Immune Tumor Microenvironment and Its Clinical Ramifications in Gynecologic Malignancies

**DOI:** 10.3389/fendo.2018.00297

**Published:** 2018-06-05

**Authors:** Muna Alemi Yahya, Shilhav Meisel Sharon, Shay Hantisteanu, Mordechai Hallak, Ilan Bruchim

**Affiliations:** ^1^Gynecologic Oncology Division, Department of Obstetrics and Gynecology, Hillel Yaffe Medical Center, Hadera, Israel; ^2^Gynecology Laboratory, Department of Obstetrics and Gynecology, Hillel Yaffe Medical Center (Affiliated with the Technion Israel Institute of Technology), Hadera, Israel; ^3^Department of Human Molecular Genetics and Biochemistry, Sackler School of Medicine, Tel Aviv University, Tel Aviv, Israel

**Keywords:** immunotherapy, ovarian cancer, cervical cancer, endometrial cancer, gynecologic cancers, insulin-like growth factor 1 pathway, insulin-like growth factor 1 receptor, targeted therapy

## Abstract

Treatment of patients with gynecologic malignancies diagnosed at advanced stages remains a therapeutic challenge. Survival rates of these patients remain significantly low, despite surgery and chemotherapy. Advances in understanding the role of the immune system in the pathogenesis of cancer have led to the rapid evolution of immunotherapeutic approaches. Immunotherapeutic strategies, including targeting specific immune checkpoints, as well as dendritic cell (DC) immunotherapy are being investigated in several malignancies, including gynecological cancers. Another important approach in cancer therapy is to inhibit molecular pathways that are crucial for tumor growth and maintenance, such as the insulin-like growth factor-1 (IGF1) pathway. The IGF axis has been shown to play a significant role in carcinogenesis of several types of tissue, including ovarian cancer. Preclinical studies reported significant anti-proliferative activity of IGF1 receptor (IGF1R) inhibitors in gynecologic malignancies. However, recent clinical studies have shown variable response rates with advanced solid tumors. This study provides an overview on current immunotherapy strategies and on IGF-targeted therapy for gynecologic malignancies. We focus on the involvement of IGF1R signaling in DCs and present our preliminary results which imply that the IGF axis contributes to an immunosuppressive tumor microenvironment (TME). For the long term, we believe that restoring the TME function by IGF1R targeting in combination with immunotherapy can serve as a new clinical approach for gynecological cancers.

## Introduction

### Treatment of Gynecologic Malignancies: Current Advances

Endometrial cancer is the most common gynecological malignancy in the developed world and the second most common in developing countries (1, 2). Treatment includes surgery and adjuvant radiotherapy, and/or chemotherapy depending on surgical staging. Women with metastatic or recurrent disease have poor prognosis, with median survival of 7–12 months (3). Endometrial cancer has two histopathological subtypes. About 80% of endometrial cancers are Type I, which has several mutations, including microsatellite instability (MSI), KRAS, PTEN, PIK3CA, and β-catenin. Type II, usually aneuploidy with poorer prognosis, contains alterations in p53, HER2/neu, p16, E-cadherin CDKN2A, and/or ERBB2 genes. Patients with mismatch repair-deficient (dMMR) or MSI tumors who have progressed on platinum-based chemotherapy might be particularly sensitive to immune checkpoint inhibitors, such as pembrolizumab, a humanized antibody that targets the PD-1 receptor (described in more detail in Section Author’s Perspective). In addition, the mTOR inhibitors everolimus and temsirolimus demonstrated antitumor activity in endometrial cancer, with greatest sensitivity in cells with *PIK3CA* or *PTEN* mutations (4, 5). Another important agent is bevacizumab, a monoclonal antibody against vascular endothelial growth factor (VEGF), which was shown to be effective in endometrial cancer (6). Other targeted therapies against somatic mutation in endometrial cancer, including PI3K and MEK, are under investigation (7–9).

Cervical cancer is the third most common cause of death from gynecological malignancies in the United States (1). The pathology behind cervical cancer is related to human papilloma virus (HPV) infection, especially genotypes 16 and 18. This finding led to the development of vaccines to prevent HPV infection. Despite the known etiology and the PAP screening test, advanced cervical cancer is a common diagnosis. The standard treatment of advanced cervical cancer is based on chemotherapy; however, poor survival rates have led to new therapeutic approaches. Recent Phase 3 studies found that adding bevacizumab to standard chemotherapy improved overall survival and progression-free survival in women with advanced, metastatic, or recurrent cervical cancer (10). Other immunotherapeutic models aimed at targeting the E6 and E7 oncoproteins of HPV will be discussed in Section “Author’s Perspective.”

Ovarian cancer is the second most common cancer and the leading cause of death from gynecological malignancy in the United States (2, 11). Epithelial ovarian cancer (EOC) represents approximately 90% of ovarian cancers. Conventional treatment includes surgical cytoreduction and adjuvant chemotherapy, which may lead to recovery in early stages. Unfortunately, there are no efficient screening tests to enable early diagnosis; hence, the vast majority of patients are diagnosed at an advanced stage and 80% of these patients will have recurrence and ultimately die of the disease (12–14). Consequently, intensive research has been undertaken to investigate alternative therapies for this disease. Angiogenesis plays a fundamental role in the pathogenesis of EOC; therefore, bevacizumab is used as an adjuvant therapy, as it prolongs progression free survival and may improve overall survival in high-risk patients (15–18). Additional agents are the poly ADP-ribose polymerase (PARP) inhibitors, which inhibit the PARP protein that functions in single strand DNA repair, leading to apoptosis. The PARP inhibitors are most effective in cancers with a BRCA mutation, because BRCA protein is involved in double-stranded DNA repair (19). Olaparib, a PARP inhibitor agent, is currently approved in the USA and Europe for patients with recurrent, platinum-sensitive, BRCA-mutation ovarian cancer (11, 20). Nowadays, precision medicine is getting more attention in the field of gynecology-oncology. Barroilhet and Matulonis provides an updated overview regarding this concept which is based on tumor gene sequencing, in order to match agents targeted against specific tumor mutations regardless of the involved organ (21).

### Immunotherapeutic Approaches for Gynecological Cancers

The immune system is composed of humoral and cellular immune responses. Cell-mediated immunity is important for eliminating cells infected with pathogen and tumor cells; the dendritic cells (DCs) are professional antigen-presenting cells (APCs) that express pattern recognition receptors. These receptors together with cytokines and chemokines cause peripheral immature DCs to mature and migrate to lymphoid tissue, where they interact with lymphocytes (22–25). The humoral response is mediated by antibodies against pathogens. As antigens enter the body, B cells respond by undergoing activation, proliferation, and differentiation to release antibodies (26). The formation of antigen-specific antibodies requires B and T lymphocytes, as well as APCs. Based on the immuno-editing concept (27), the immune system eradicates new emerging tumor cells; however, in some cases one cell remains dormant, escapes the immune system, and proliferates leading to disequilibrium between the immune system and cancerous cells. Immune-inflammatory cells, among others, comprise the tumor microenvironment (TME). Considering the widely established link between inflammation and cancer, the TME has a significant role in tumorigenesis (28–31). Consequently, immunotherapeutic approaches against cancer have recently emerged.

The main immunotherapeutic approaches are DC vaccines and blockade of immune checkpoints, including programmed cell death, PD-1/PD-L1. PD-1 is an immunoinhibitory receptor that is expressed on T cells, B cells, monocytes, natural killer cells, and many tumor-infiltrating lymphocytes. Interaction of the receptor with its ligand, the PD-L1, which was found to be expressed in some cancer cells, restrains the immune system from attacking cells in the body (32, 33). The immune system’s involvement in endometrial cancer is not fully understood; the FDA approved pembrolizumab (anti PD-1 antibody) for patients with MSI or MMR deficiency who did not respond to prior therapies (34).

Cervical cancer is related to chronic HPV infection in 99.7% of cases. The HPV integrates into the cellular genome and expresses two oncoproteins, E6 and E7. The E6 oncoprotein inhibits p53; thus, leading to proliferation, while E7 prevents apoptosis (35). Immunotherapeutic approaches which target the E6 and E7 oncoproteins are under investigation. Therapeutic vaccines which include vaccinia- and listeria-based vaccines and DC vaccines have shown promising results (35, 36). Specifically, DC vaccines, which are based on autologous DCs transfected with E6 or E7 RNA in order to stimulate cytotoxic T cell response, led to effective eradication of cervical cancer cells (37). Unfortunately, small clinical trials demonstrated T cell response without a clinical benefit (38, 39). The immunoinhibitory ligand, PD-L1 was observed in 95% of cervical intraepithelial neoplasia and in 80% of cervical squamous cell carcinoma, but was not detected in normal cervical epithelial cells implying a potential therapeutic benefit (40). In the KEYNOTE-028 trial, pembrolizumab has shown preliminary promise as second-line therapy for cervical cancer (41). Finally, CAR-T cells are genetically modified T cell that are taken from tumor tissue, expanded *ex vivo* and transferred back into the patient. A small trial on nine patients with recurrent metastatic cervical cancer showed a complete response in one patient and partial response in two others (42).

Despite significant advances in surgery and chemotherapy treatments, ovarian cancer is still the most lethal of all gynecological malignancies. Data suggest that the presence of tumor-infiltrating lymphocytes at diagnosis, improves survival rates (43, 44). This reflects the crucial role of the immune system in eliminating tumor cells. Of interest, early reports showed significant efficacy for the CAR-T strategy (45). Treatments targeting the immune checkpoints PD-1/PD-L1 and the inhibitory receptors of cytotoxic T lymphocyte-associated antigen 4 were approved for use in melanoma and have been evaluated for treatment of EOC (46–48). The anti-PD-1 agent nivolumab, and the anti PD-L1 agents avelumab and pemrolizumab, are being evaluated in clinical trials with promising results (48–50). Various vaccine models, including simple vaccine preparations consisting of proteins expressed in EOC and more complex models, such as DC vaccines have been developed. In the latter model, DCs are matured outside the body and programmed to detect and attack tumor cells upon reinjection into the patient (51).

### The Insulin-Like Growth Factor (IGF) Axis in Gynecological Cancer

The IGF system has a pivotal role in cell proliferation, differentiation, and apoptosis (52). This system is composed of ligands (IGF-1, IGF-2), IGF receptors (IGF1R, IGF2R), and six IGF binding proteins (IGFBPs 1–6). The biologic effects of the ligands are mediated by the IGF1R, which undergoes autophosphorylation of its tyrosine kinase domain, with ensuing phosphorylation of insulin receptor (INSR) substrates. Consequently, the ras–raf–MAP kinase and the PI3K–PDK1–Akt/PKB signaling pathways are activated, resulting in metabolic actions, proliferation, and reduction in apoptosis (53). Unlike the IGF1R, the IGF2R, which is apparently not involved in IGF signaling, is mainly responsible for targeting the highly mitogenic IGF2 for lysosomal degradation (54). Worthy of mention, the IGF1R shares a high degree of homology with the INSR, leading to a certain level of cross talk between insulin, IGFs, and their receptors. The complexity of these interactions was widely discussed and studied by several studies (55–58). The interplay between the IGF signaling and estrogen pathways is of importance as well (59), and relevant to us is the well-established cross talk between these pathways in endometrial cancer (60, 61). In addition to its normal physiological roles, the IGF axis and in particular, the IGF1R are also involved in carcinogenesis. Epidemiologic observations showed an association between circulating IGF-1 levels and prostate, premenopausal breast and colorectal cancer incidence (62). Consequently, the IGF axis became an attractive therapeutic target.

Targeting IGF1R with specific monoclonal antibodies inhibited IGF-induced proliferation in both Type I and II endometrial cancer (63, 64). Moreover, a human monoclonal IGF1R antibody inhibited endometrial cancer proliferation in clinical trials (65). In cervical cancer animal models, treatment with IGF1R antibodies inhibited tumor growth and caused tumor regression (66). In addition to monoclonal antibodies, small molecule IGF1R inhibitors such as NVP-AEW541 had anti-proliferative effects on ovarian cancer cells (67). Current ovarian cancer clinical trials are evaluating IGF inhibitors alone, or in combination with biologic agents or chemotherapy.

These findings and others have turned the IGF1R into a promising target for treating cancer; yet there is no clear-cut evidence to approve its use as a single agent.

## Author’s Perspective

The complexity of the IGF1R signaling pathway was suggested as one cause of the failure of IGF-targeting strategies (68). Parallel growth and survival pathways, as well as a lack of proper biomarkers for patient selection are additional possible explanations. Given the genetic complexity of most tumors, including gynecological cancers, accumulating evidence suggests that combination strategies of targeting multiple signaling pathways will probably be required to obtain therapeutic effects. Shao et al. showed that dual anti-VEGF and anti-IGF1R treatment (bevacizumab and cixutumumab, respectively) enhanced tumor growth inhibition in ovarian cancer cells and provided significant benefit over either treatment alone (69). Another study reported that the IGFIR kinase inhibitor BMS-536924 increased the cytotoxicity induced by the PARP inhibitor 3-aminobenzamide in human EOC cell lines, suggesting a combination of IGF1R inhibitor and a PARP inhibitor to decrease resistance to treatment (70). Haluska et al. demonstrated functional cross talk between the IGF and HER family of receptors in ovarian cancer cells (71). In line with the development of immunotherapeutic approaches in various cancer types, targeting the IGF1R signaling pathway in combination with immunotherapy should be investigated. This is supported by the finding that PI3K–AKT pathway inhibitors sensitize tumor cells to immunotherapy (72). It seems that a deep understanding of the involvement of the IGF pathway in host immunity and in immune cells in the TME constitutes a basis for the combination targeting strategy described above. Interestingly, a recent published review describes a similar approach regarding the estrogen pathway involvement in TME (73). In the context of combined therapies estrogen is highly relevant, considering the interplay between estrogen and IGF1. The role of IGF1R in the development and function of the immune system appears to be complex and variable; nonetheless, there is compelling evidence that within a TME, the IGF axis promotes an immunosuppressive, anti-inflammatory response that enables cancer growth. For example, IGF1 was shown to negatively regulate DC activation; thereby impairing the antigen-presentation function (74). IGF1 was also shown to stimulate the proliferation of Treg cells, which are known to suppress local T cell immune responses (75). Moreover, IGF1R activation was linked to macrophage polarization from the pro-inflammatory M1 to the pro-tumorigenic M2 phenotype in various animal models (76). A recent study showed that IGF1 production by myeloid-derived suppressor cells promoted stromal cell migration and tumor invasion, implying that IGF1 might also play a role in the tumor-promoting effect of myeloid-derived suppressor cells (77). Taken together, the interplay between the IGF1 axis and immune cells should be investigated further. This will contribute to future studies involving IGF1R targeting in combination with immunotherapy for gynecologic malignancies.

### IGF1R Monoclonal Antibodies and Tyrosine Kinase Inhibitor (TKI) Inhibit the IGF1-Induced Activation of Intracellular Cascades

We have been investigating the effect of IGF1R targeting in endometrial and ovarian cancer for several years. IGF1R-targeting with monoclonal antibodies and specific IGF1R TKIs inhibited IGF-induced proliferation in both Type I and II endometrial carcinomas (63, 64). In addition to the eliminated IGF1-stimulated proliferation, IGF1R inhibitors increased apoptosis. Cixutumumab, a fully human monoclonal antibody against IGF1R, inhibited IGF1-mediated biological actions and cell signaling events in four endometrial carcinoma-derived cell lines. Cixutumumab blocked the IGF1-induced autophosphorylation of IGF1R and reduced IGF1R expression. Recent studies demonstrated that MK-0646 had a potent anti-proliferative effect in Type I and II endometrial cancer cell lines, associated with a decrease in IGF1-induced IGF1R, AKT, and ERK1/2 phosphorylation. Interestingly, a different response to IGF1R blocking with MK-0646 was observed in Type I and Type II endometrial cancer. In addition, a previous study showed that IGF1R-targeted therapy has significant anti-neoplastic activity in ovarian cancer cells (67).

### Tumor Suppressor p53 and BRCA1 Are Potential Biomarkers for IGF1R-Targeted Therapy

Following the failure of the IGF-targeting strategies, another possible approach is identifying predictive tumor biomarkers that will increase the efficacy of IGF1R-targeted therapy. These predictive biomarkers are intended for the process of developing early, innovative, patient screening methodologies that will predict the response to personalized therapy and resistance to IGF1R-targeted therapy. Several studies have provided evidence that the IGF1R gene transcription rate depends on a number of stimulatory nuclear proteins and is modulated by negative transcriptional regulators, including p53, p63, and p73 (78, 79) and the BRCA1 gene (80, 81). The IGF1R system is regulated by the p53 pathway in several malignancies, including endometrial cancer and ovarian cancer (82). Using USC-derived cell lines, Attias-Geva et al. demonstrated that p53 negatively regulates IGF1R gene expression *via* a mechanism that involves interaction with the zinc finger protein Sp1, a potent transactivator of the IGF1R gene (82). BRCA1 is a tumor suppressor whose mutation was correlated with the appearance of familial breast and/or ovarian cancer at young ages. Our group reported a high rate (25.8%) of the predominant BRCA1/2 mutations in unselected Jewish patients with USC (83). Moreover, immunohistochemical studies of USC tumors revealed high protein expression of BRCA1 and IGF1R in primary and metastatic tumors. Interestingly, we found that BRCA1 expression led to significantly reduced IGF1R promoter activity in USC cell lines. These results are consistent with the notion that loss of inhibitory control due to BRCA1 mutation may lead to enhanced IGF1R expression and eventually, increased proliferation (84). Taken together, our results suggest that BRCA1 mutational status may predict IGF1R inhibitor efficacy. This is supported by the recent study of Cohen-Sinai et al., which demonstrate a reduced inhibitory effect of anti-IGF1R treatment in mutant BRCA1-expressing cells (85).

### Involvement of the IGF Axis in DCs

**Figure 1:**
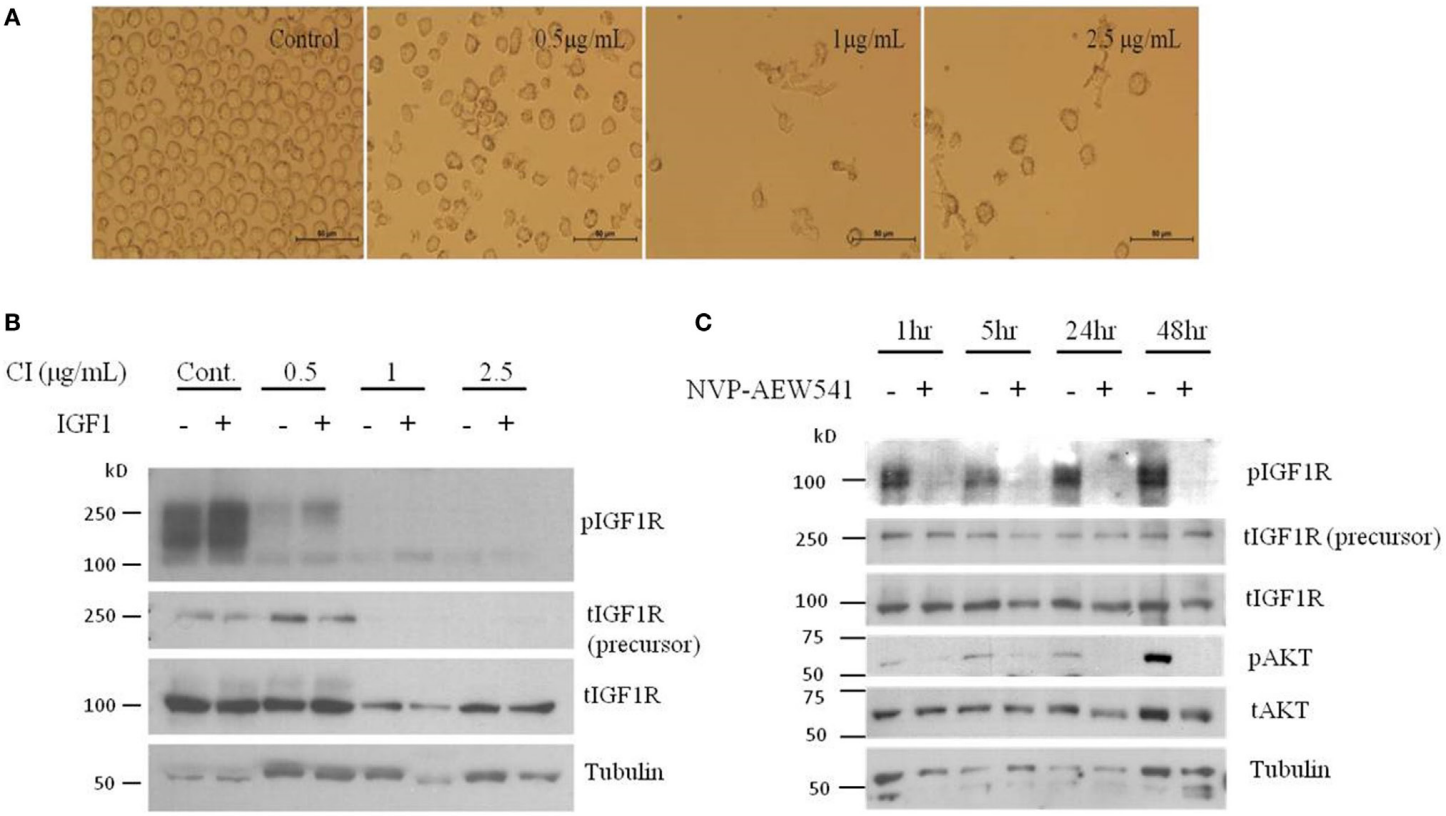
Dendritic cell (DC) differentiation of HL-60 cells show reduced total and phosphorylated IGFIR protein levels. **(A)** Human leukemic HL-60 cells were differentiated to DCs by treatment with 0.5, 1, and 2.5 μg/ml of calcium ionophore (CI) for 72 h. **(B)** Human leukemic HL-60 cells were treated with 0.5, 1, and 2.5 g/ml of CI for 72 h and 10 min before harvest, cells were treated with insulin-like growth factor (IGF)1. Whole-cell lysates were resolved on SDS-PAGE and immunoblotted with the specified antibodies. Level of tubulin was used as a loading control. **(C)** Human leukemic HL-60 cells were treated with 2 μM of NVP-AEW541 for 1, 5, 24, and 48 h. Whole-cell lysates were resolved on SDS-PAGE and immunoblotted with the specified antibodies. Level of tubulin was used as a loading control.

**Figure 2:**
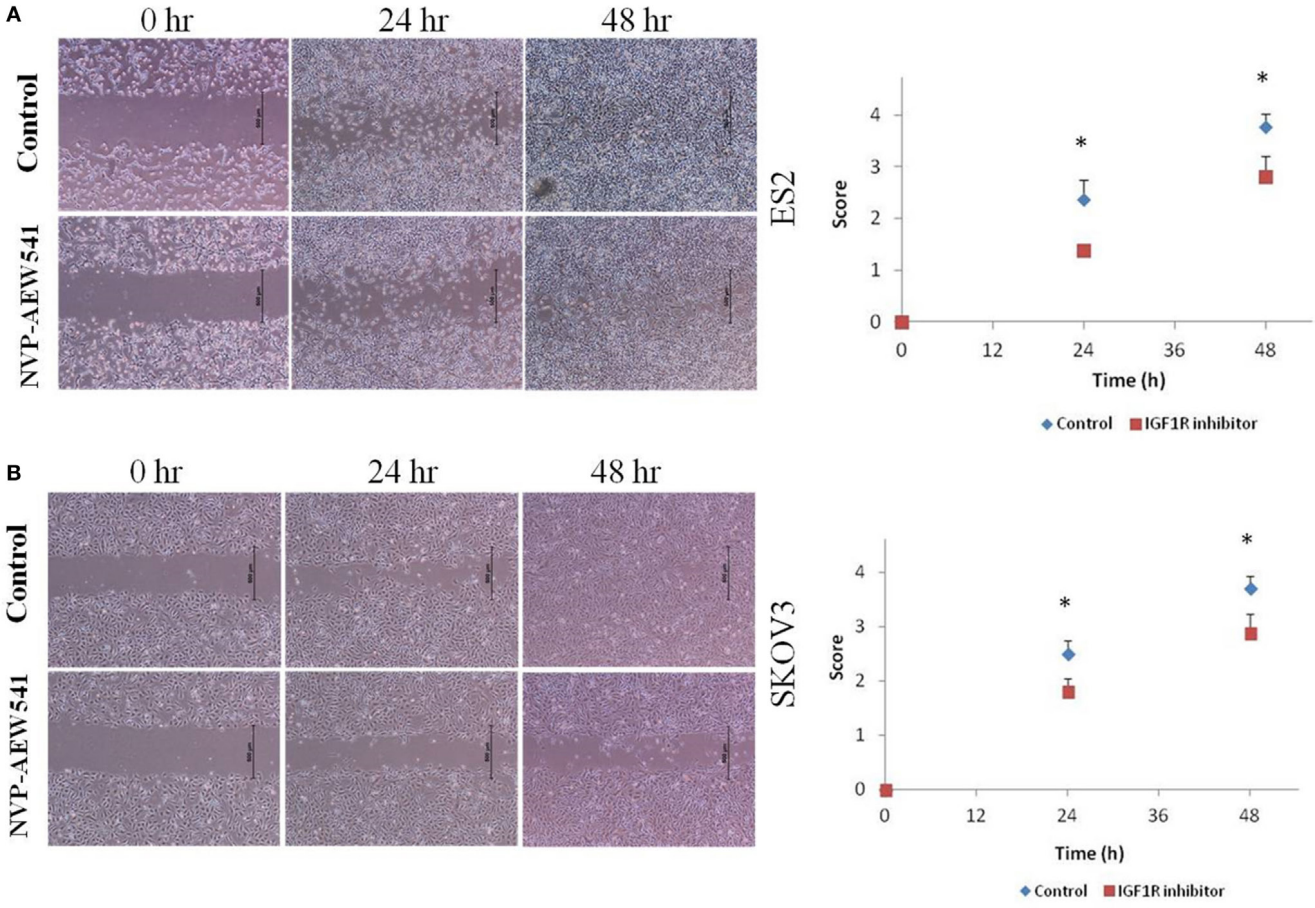
NVP-AEW541 treated dendritic cells decrease ovarian cancer cell migration. Cell migration was detected by wound scratch assay. Representative wound closure images from three experiments are shown. Human leukemic HL-60 cells were treated with 1 μg/ml of CI and with 2 μM of NVP-AEW541 for 72 h after which they were co-cultured with the epithelial ovarian cancer (EOC) cells ES2 **(A)** and SKOV3 **(B)**. Scratch was applied 24 h post cell merge. The growth of EOC cells into the scratch zone is demonstrated here at time 0, 24, and 48 h after scratch. The graphs represent average growth score of three independent experiments of ES2 and SKOV3 cells (*P < 0.05).

Although the involvement of the IGF axis and the IGF1R in ovarian cancer has been widely investigated, the exact function of IGF1R in host immunity and in tumor-infiltrating immune cells is still not clear. It has been shown that IGF1 is expressed in many immune cells and bone marrow stromal cells (86). In addition, nearly all immune cells such as T lymphocytes and B lymphocytes (87), mononuclear cells (88), and NK-cells (89) express IGF1R and are susceptible to the effects of IGF. A recent study showed that long-term IGF treatment resulted in delayed maturation of DCs and suppression of DC-mediated immunity. Specifically, treatment of DCs with the IGF1R inhibitor NVP-AEW541 restored DC-mediated antigen presentation and antitumor immunity (90). Moreover, it has been shown that IGFs enhance the secretion of IL-10 in bone marrow monocyte-derived DCs, thereby enhancing the immunosuppressive status of the tumor environment. Accordingly, blocking the IGF1 signaling pathway, apart from its effect on cancer cells, provides a new target to generate potent antitumor immunity by rescuing the impaired function of DCs. To examine IGF1R signaling activation in DCs, human leukemic HL-60 cells were differentiated to DCs using calcium ionophore (Figure 1A), treated with IGF1 and harvested, followed by western blot assay. Results show that DC differentiation was associated with a marked decrease in phosphorylated levels of IGF1R and in total IGF1R expression (Figure 1B). Of interest, undifferentiated HL-60 cells displayed high basal levels of phosphorylated IGF1R. Since undifferentiated HL-60 cells (control) exhibited basal phosphorylation of IGF1R, cells were treated with the IGF1R inhibitor prior to DC differentiation. To examine the effect of IGF1R inhibition on the IGF1 pathway in HL-60 cells, the cells were treated with IGF1R inhibitor (NVP-AEW541) for 1, 5, 24, and 48 h. As a result, IGF1R and AKT (downstream mediator) phosphorylation levels decreased (Figure 1C). Considering these results, it was then relevant to question whether blocking IGF1R in DCs would have a significant effect on ovarian carcinoma cells. DC differentiation was induced with or without NVP-AEW541 treatment. Differentiated cells were then co-cultured with EOC cells (ES2 and SKOV3) and a wound scratch assay was performed. As shown in Figure 2, results demonstrated decreased migration of ES2 and SKOV3 cells when co-cultured with DCs pre-treated with NVP-AEW541, as compared to untreated DCs. To note, an initial flow cytometry experiment implies that the NVP-AEW541 treatment accelerates the DCs maturation (not shown). Nonetheless, our findings are preliminary and should be furtherly studies.

## Summary and Conclusion

Collectively, recent evidence suggests that in addition to its direct role in tumor cell growth, the IGF axis contributes to the immunosuppressive TME leading to inhibition of the antitumoral T-cell-mediated responses. It is expected that immunotherapy will be potentiated when combined with IGF1R signaling blockade directed at inactive antigen-presenting DC or suppressive immune cells. Future studies evaluating the role of the IGF1 signaling pathway in tumor infiltrating immune cells in gynecologic malignancies will deepen our understanding of the feasibility of the combined therapy. Moreover, characterizing the TME and identifying new biomarkers will contribute to the development of precision gynecologic cancers treatments.

## Author Contributions

MY and SS designed and performed the laboratory studies presented in the manuscript. MY and SS acquired and interpreted the data presented in the manuscript. MY and SS were involved in writing the drafts of the paper and the final version. SH was involved in the designing and performing the laboratory studies presented in the manuscript. He was involved in writing the drafts of the paper and the final version. MH was involved in the designing of the laboratory studies presented in the manuscript. He was involved in writing the drafts of the paper and the final version. IB designed the studies, acquired and interpreted the data presented in the manuscript. He was involved in writing the drafts of the paper and the final version. Also in submitting the manuscript.

## Conflict of Interest Statement

The authors declare that the research was conducted in the absence of any commercial or financial relationships that could be construed as a potential conflict of interest.
