# Supplementary material for: The Role of the Insulin-Like Growth Factor 1 Pathway in Immune Tumor Microenvironment and Its Clinical Ramifications in Gynecologic Malignancies
Source: Front Endocrinol (Lausanne). 2018 Jun 5;9:297. doi: 10.3389/fendo.2018.00297 (PMC5996273; doi:10.3389/fendo.2018.00297)
Supplement: Supplementary file 1 [file data_sheet_1.docx]

APPENDIX METHODS

**Cell cultures**

HL-60, a human leukemic cell line, was provided by I P. Witz's laboratory (Tel-Aviv University) and cultured in RPMI 1640 medium supplemented with 10% fetal bovine serum (FBS). SKOV3 (serous carcinoma) and ES2 (clear cell carcinoma) human ovarian cancer cell lines were obtained from I. Tsarfaty's laboratory (Tel-Aviv university) and cultured in DMEM supplemented with 10% FBS, 2 mM glutamine and 100 units/ml penicillin + 100 mg/ml streptomycin. All cell lines were maintained at 37°C with 5% CO_2_. All reagents were purchased from Biological Industries Ltd., Kibbutz Beit Haemek, Israel.

**Cell treatments**

Differentiation of HL-60 leukemic cells to DCs was induced by Calcium Ionophore (CI) A23187 (Sigma-Aldrich Co., St. Louis, MO, USA) treatment.

Ten minutes before harvest, cells were treated with 50 ng/ml of IGF1 (Cytolab Ltd., Rehovot, Israel).

HL-60 and DCs were treated with 2 µM of NVP-AEW541 (Novartis Pharma, Basel, Switzerland) in a time-dependent manner.

**Western blot analyses**

Cells were lysed in a protease-inhibitor containing buffer. Samples were electrophoresed through 10% SDS-PAGE, followed by blotting of the proteins onto nitrocellulose membranes. After blocking with 5% skim milk or 3% bovine serum albumin (BSA), the blots were incubated overnight with the indicated antibodies, washed, and incubated with the appropriate horseradish peroxidase (HRP)-conjugated secondary antibody.

**Wound scratch assay**

NVP-AEW541 (a selective IGF1R inhibitor) treated HL-60 cells were differentiated into DCs. ES2 and SKOV3 cells were seeded in 6 well plates and 24 hr after seeding co-cultured with the pretreated differentiated HL-60 cells. Thereafter two scratches were applied in each well with a sterile 200µl pipette tip. Ovarian carcinoma cell growth into the scratch area was followed under a microscope and image was captured every 24 hr.
